# High-frequency 10 kHz Spinal Cord Stimulation for Chronic Back and Leg Pain: Cost-consequence and Cost-effectiveness Analyses

**DOI:** 10.1097/AJP.0000000000000866

**Published:** 2020-08-04

**Authors:** Rod S. Taylor, Anthony Bentley, Bruce Campbell, Kieran Murphy

**Affiliations:** * Institute of Health and Well Being, University of Glasgow, Glasgow; † Institute of Health Research, University of Exeter Medical School; § University of Exeter Medical School, Exeter; ‡ Mtech Access Limited, Bicester, Oxfordshire, UK; ∥ Nevro Corporation, Redwood City, CA

**Keywords:** spinal cord stimulation, economic evaluation, back and leg pain, cost-utility

## Abstract

There is good evidence that spinal cord stimulation (SCS) is effective for reducing chronic back and leg pain (CBLP). SENZA randomized controlled trial showed high-frequency (10 kHz) stimulation (10 kHz-SCS) is clinically superior to traditional low-frequency SCS (LF-SCS).

Undertake cost-consequence and cost-effectiveness analysis of 10 kHz-SCS compared with LF-SCS.

**Methods:**

A probabilistic decision tree and Markov decision analytic model was used to synthesize data on CBLP outcomes and costs over a 15-year time horizon from a UK National Health Service perspective using data from the SENZA randomized controlled trial and other publications. Results are expressed as incremental cost per quality-adjusted life year (QALY) in 2016 Pounds Sterling.

**Results:**

10 kHz-SCS is cost-saving and cost-effective compared with LF-SCS, with mean cost-savings of £7170 (95% confidence interval: £6767-£7573) and £3552 (95% confidence interval: £3313-£3792) per patient compared with nonrechargeable and rechargeable LF-SCS devices, respectively. 10 kHz-SCS has a 95% likelihood of being cost-effective at a willingness-to-pay threshold of £20,000 per QALY. Our findings were robust across a wide range of sensitivity analyses.

**Conclusions:**

There is a strong economic case for choosing 10 kHz-SCS over LF-SCS for CBLP. Furthermore, 10 kHz-SCS has clinical advantages not captured in our analysis, including shorter, and more predictable procedure times.

## Abstract 2

Supplemental Digital Content is available in the text.

Chronic back and leg pain (CBLP) is a major societal burden, ranking first for disability and sixth for overall disease burden.^1^ Total annual expenses related to CBLP are ~£12 billion in the United Kingdom and over $100 billion in the United States.^2^,^3^ CBLP causes considerable debility, and a growing number undergo spinal surgery. Some 20% of those who receive back surgery nevertheless continue to experience CBLP—often known as failed back surgery syndrome (FBSS).^3^

Spinal cord stimulation (SCS) has been shown to reduce back and leg pain, improve functional capacity and enhance health-related quality of life (HRQoL).^4^,^5^ It is recommended in clinical guidelines for both CBLP and FBSS patients when conventional medical management (CMM) fails to control symptoms.^6^–^8^ SCS involves the implantation of a stimulation device connected to electrodes placed near the spinal cord. It can be delivered in several different ways by a range of different devices. Historically, stimulation frequencies of 50 to 80 Hz have been used (low-frequency stimulation [LF-SCS]), delivered by devices with nonrechargeable batteries which need to be replaced surgically when they deplete, typically 1 to 4 years after initial implantation.

The SENZA randomized controlled trial (SENZA-RCT) allocated 198 participants with CBLP across 10 US centers to either 10 kHz high-frequency SCS (10 kHz-SCS, Senza System, Nevro Corp, Redwood City) or traditional LF-SCS, (Precision Plus SCS system, Boston Scientific Corporation, Marlborough, MA).^9^ At 3 months, 84.5% of 10 kHz-SCS patients with back pain and 83.1% with leg pain were responders (≥50% pain reduction with no stimulation-related significant adverse events [AEs]) or increase in opioids, compared with 43.8% and 55.5%, respectively who received LF-SCS (*P*<0.001 for both comparisons). The superiority of 10 kHz-SCS over LF-SCS for back and leg pain was maintained at 12- and 24-month follow-up.^9^,^10^

In 2015, the US Food and Drug Administration (FDA) granted regulatory approval for the 10 kHz-SCS device based on the SENZA-RCT and approved a label which confirmed superiority over LF-SCS.^11^

Regulatory approval (based on evidence of safety, performance, and efficacy) is only the initial step in the adoption of any medical device by health systems. Worldwide, health care policy makers and payers are faced with funding challenges and they need evidence of cost-effectiveness or “value for money” of new health technologies. Economic analyses have consistently shown SCS to be highly cost-effective for the treatment of CBLP and FBSS^12^ with a reported incremental cost effectiveness ratio (ICER) of SCS compared with CMM or reoperation well below a maximum willingness to pay (WTP) threshold of £20,000 to £30,000 (or currency equivalent) per quality adjusted life year (QALY)—the threshold commonly used by policy makers in various developed health care economies.^13^ One economic study specifically considered 10 kHz-SCS, reporting that it showed a highly favourable ICER over a 15-year time horizon of £3153 per QALY gained compared with CMM; and dominance (less costly, more QALYs) compared with LF-SCS.^14^ However, as the authors acknowledged, this analysis was limited by use of non-randomized controlled trial (RCT) data and an indirect comparison between 10 kHz-SCS and LF-SCS.

In January 2019, the UK’s National Institute of Health and Care Excellence (NICE) Medical Technologies Guidance (MTG41) recommended 10 kHz-SCS for chronic neuropathic back or leg pain,^15^ based on an assessment of clinical effectiveness evidence and a cost-consequence analysis.^16^ In this analysis, we provide previously unpublished details of the cost-consequence analysis that underpinned NICE’s assessment. In addition, we present a new cost-effectiveness analysis of 10 kHz-SCS compared with LF-SCS.

## METHODS

This economic evaluation is reported in accord with the Consolidated Health Economic Evaluation Reporting Standards (CHEERS) statement^17^ and based on NICE reference methods.^18^

### Study Design

This economic analysis was undertaken from the perspective of the UK National Health Service using data from the SENZA-RCT.^9^,^10^ We reproduced the economic model used in the 2008 NICE Technology Appraisal of SCS.^8^ Comparisons were made separately for nonrechargeable (NRLFSCS) and rechargeable (RLF-SCS) variants of low-frequency SCS devices (LF-SCS).

The model simulates a population of adult patients (18 y and above) with CBLP (with pain intensity score of ≥5 cm on visual analogue scale [VAS]) despite CMM.^9^,^10^ A time horizon of 15 years and a discount rate of 3.5% were used for both costs and outcomes.^8^,^16^ The time horizon reflects the chronic nature of the condition and the intended longevity of the devices, ensuring at least one replacement procedure for each device type (10 kHz-SCS, NRLF-SCS, and RLF-SCS).

### Model Structure

**FIGURE 1:**
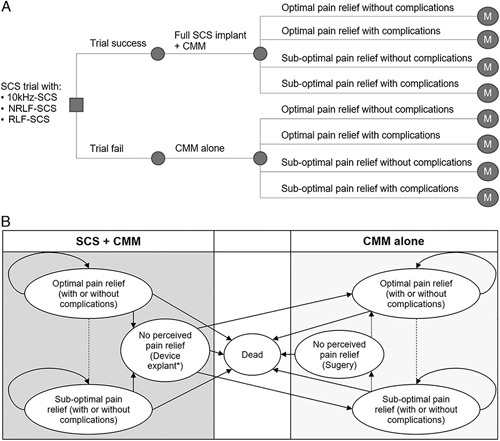
Six-month decision tree (A) and long-term Markov model schematics (B). *SCS devices may also be removed due to paresthesia and other adverse events. 10 kHz-SCS indicates 10 kHz high-frequency spinal cord stimulation; CMM, conventional medical management; M, Markov model; NRLF-SCS, nonrechargeable low-frequency spinal cord stimulation; RLF-SCS, rechargeable low-frequency spinal cord stimulation; SCS, spinal cord stimulation.

The model was developed in Microsoft Excel 2016 comprising a decision tree and Markov “state transition” model. On the basis of relevant clinical data, the decision tree was used to explore the clinical pathway of patients in the short-term (first 6 mo) after SCS implantation (Fig. 1A), and the Markov model used over the long-term (Fig. 1B).

Before entering the decision tree, all patients allocated to SCS first undertook a SCS screening trial, with an external stimulator for up to 14 days to assess pain relief, as per clinical practice. Patients with satisfactory pain relief (≥50% reduction in VAS score), received a permanent SCS implant and those without sufficient relief received CMM alone. After permanent implantation, the decision tree considered the initial 6 months’ response to treatment with or without complications (Fig. 1A). After 6 months, patients entered a Markov model to explore the clinical pathways over the long-term (Fig. 1B) using 6 mutually exclusive health states:

1. Optimal pain relief (defined as ≥50% reduction in VAS score for leg pain) with no complications.
2. Optimal pain relief with complications (device related complications, eg, lead migration or other complications, eg, infections).
3. Sub-optimal pain relief (some pain relief but <50% reduction in VAS score for leg pain) with no complications.
4. Sub-optimal pain relief with complications.
5. No perceived pain relief (no impact on pain experienced by the patient despite a well-functioning device).
  (a) Consequently, this relates to a change in treatment strategy (eg, device explant/removal or subsequent reoperation) and reverting to CMM alone.
  (b) Patients may also have a device explant due to intolerable paresthesia or other complications (eg, surgical site infection).
6. Death (all-cause mortality).

During each 3-month cycle, SCS patients’ health state remained unchanged (optimal or sub-optimal pain relief), unless they:

1. Had their SCS device removed due to insufficient pain relief, intolerable paresthesia or other complication (eg, infection).
2. Received spinal surgery for insufficient pain relief (CMM alone arm).
3. Died.

Patients with optimal or sub-optimal pain relief, could experience device-related complications not requiring a device explant in each cycle. All-cause mortality was included but no device or procedure-related deaths were modelled.

### Clinical Inputs and Model Parameters

Modelling assumptions (reported in Table 1), model structure and health state definition are consistent with the 2008 NICE Technology Appraisal.^8^ The clinical data used in the decision tree (first 6 mo) for trial success and optimal pain relief (≥50% reduction in leg pain from baseline) in the base-case was taken from the SENZA-RCT.^9^,^10^ Probabilities for optimal pain relief with or without complications, and sub-optimal pain relief with or without complications were calculated from the SENZA-RCT. Complications, included in the model, were AEs not resulting in a device explant (including implant site pain, surgical site infection, delayed wound healing, paresthesia, lead migration, and device dislocation) and were derived from patient-level analysis of the SENZA-RCT. Probabilities for optimal pain relief without complications, optimal pain relief with complications, sub-optimal pain relief without complications and sub-optimal pain relief with complications were also derived from the SENZA-RCT. The base-case values used in the model are outlined in Table 1. In the Markov model, long-term complication rates and device explant rates were based on an analysis of patient-level data from the SENZA-RCT and obtained from the manufacturer.

**TABLE 1:** Summary of Data Inputs used in the Model

Model Parameter	Base-case Value	95% CI or Range	Source
Trial success			
10 kHz-SCS	92.8%	87.6%-97.9%	Kapural et al^9^
NRLF-SCS/RLF-SCS	88.0%	81.4%-94.7%	Kapural et al^9^
Optimal pain relief (leg pain, 6 mo)			
10 kHz-SCS	80.9%	72.7%-89.1%	Kapural et al^9^
NRLF-SCS/RLF-SCS	54.4%	43.5%-65.2%	Kapural et al^9^
CMM alone	9.3%	8.4%-10.2%	Taylor et al^19^
Non-serious complications (6 mo)			
10 kHz-SCS	33.7%	23.9%-43.5%)	SENZA-RCT, de novo analysis (source: manufacturer)
NRLF-SCS/RLF-SCS	35.8%	25.4%-46.2%)	SENZA-RCT, de novo analysis (source: manufacturer)
Annual death rate*	0.81%	0.7%-0.9%	Office of National Statistics^20^
Proportion of patients receiving a reoperation	5.0%	4.5%-5.5%	Simpson et al^21^
Proportion of patients obtaining optimal pain relief postsurgery after a reoperation	19.0%	17.1%-20.9%	Simpson et al^21^
Explant rate (Year 1)			
10 kHz-SCS	4.4%	0.2%-8.7%	SENZA-RCT, de novo analysis (source: manufacturer)
NRLF-SCS/RLF-SCS	11.1%	4.3%-18.0%	SENZA-RCT, de novo analysis (source: manufacturer)
Explant rate (Year 2)			
10 kHz-SCS	4.7%	0.2%-9.1%	SENZA-RCT, de novo analysis (source: manufacturer)
NRLF-SCS/RLF-SCS	9.7%	2.9%-16.6%	SENZA-RCT, de novo analysis (source: manufacturer)
Explant rate (Year 3)			
10 kHz-SCS	3.2%	0%-15.8%	Simpson et al^21^
NRLF-SCS/RLF-SCS	3.2%	0%-15.8%	Simpson et al^21^
Non-serious complications (beyond 6 mo)			
10 kHz-SCS	3.7%	0.6%-7.1%	SENZA-RCT, de novo analysis (source: manufacturer)
NRLF-SCS/RLF-SCS	12.8%	6.8%-18.9%	SENZA-RCT, de novo analysis (source: manufacturer)
Device longevity (y)			
10 kHz-SCS	10	8-25	Conservative assumption: 10 kHz-SCS regulatory approval has been granted for a battery life of at least 10 y of continuous use (ie, it is expected that the patient will not have to receive a new neurostimulator for at least 10 y)
RLF-SCS	10	8-25	Assumption based on review of physician manuals and previous economic evaluations
NRLF-SCS	4	2-6	Assumption based on review of physician manuals and previous economic evaluations
Utility values			
Health state			
Optimal pain relief without complications	0.598	0.538-0.658	Taylor et al^19^
Optimal pain relief with complications	0.528	0.475-0.581	Taylor et al^19^
Sub-optimal pain relief without complications	0.258	0.232-0.284	Taylor et al^19^
Sub-optimal pain relief with complications	0.258	0.232-0.284	Taylor et al^19^
No perceived pain reduction	0.168	0.151-0.185	Taylor et al^19^

*All-cause mortality (England) and assumed to be independent of health state.

10 kHz-SCS indicates 10 kHz high-frequency spinal cord stimulation; CI, confidence interval; NRLF-SCS, traditional low-frequency nonrechargeable spinal cord stimulation; RLF-SCS, traditional low-frequency rechargeable spinal cord stimulation.

Some complications require device explant and 3 broad types were identified in the SENZA-RCT, namely ineffective pain control, intolerable paresthesia, and miscellaneous other AEs (eg, surgical site infections, patient falls). Device explants were considered separately from other complications for 2 time periods: from implantation to month 6 (to reflect the decision tree time horizon) and from month 6 to the end of the 2-year study period (annualized for use in the long-term Markov model).

To identify the explant rates in Year 1 and Year 2 for 10 kHz-SCS and for NRLF-SCS/RLF-SCS devices, a de novo patient level data analysis of the SENZA-RCT was conducted.^22^ An absolute difference in the rate of explants between 10 kHz-SCS and for NRLF-SCS/RLF-SCS devices of 6.7% and 5.0% were observed in Year 1 and Year 2, respectively (Table 1). In Year 1, higher rates of explants for NRLF-SCS/RLF-SCS compared with 10 kHz-SCS were due to intolerable paresthesia, with the difference between 10 kHz-SCS therapy and NRLF-SCS/RLF-SCS in Year 2 as the result of a less effective reduction in pain with NRLF-SCS/RLF-SCS. As no 10 kHz-SCS or comparable LF-SCS data currently exists beyond Year 2 from the SENZA-RCT, we assumed that the explant rate for 10 kHz-SCS and NRLF-SCS/RLF-SCS is equivalent from Year 3 onwards, using the previously assumed explant rate of 3.2% per annum.^19^,^21^ This assumption is considered conservative (for 10 kHz-SCS) as the benefit in explant rates between 10 kHz-SCS and NRLF-SCS/RLF-SCS is not included from Year 3 onwards. We also performed a conservative analysis that assumed no difference in explant rates between 10 kHz-SCS therapy and NRLF-SCS/RLF-SCS in Year 1 and Year 2 using an explant rate of 3.2% previously assumed in the 2008 NICE model.^8^

A baseline mean device longevity of 4 years was assumed for NRLF-SCS and 10 years for 10 kHz-SCS and RLF-SCS (varied between 9 and 25 years in sensitivity analysis).

### Cost Data

Costs were sourced from the NICE 2008 appraisal and, where appropriate, inflated to 2016 GBP values using indices from Curtis and Burns^23^ to align with the assessment of clinical effectiveness evidence and a cost-consequence analysis submitted to NICE.^16^ Costs considered in the analysis included device list prices, and reimplantation, health state, AE, and additional costs.

UK prices published and referenced in peer-reviewed journals for both 10 kHz-SCS and NRLF-SCS and RLF-SCS were used. Prices were inflated using Personal Social Services Research Unit (PSSRU) Pay and Prices Index^23^ using the base year 2007/2008 for NRLF-SCS and RLF-SCS from Taylor et al^19^ and the base year 2009/2010 for 10 kHz-SCS from Annemans et al^14^ (Table 2). These prices are a “bundle price” which covers the cost of the implantation procedure and all the required consumables that could include electrodes, leads, implantable pulse generator, remote control, and battery charger.

**TABLE 2:** Summary of Cost Inputs used in the Model

Model Parameter	Base-case Value	95% CI or Range	Source
Base-case costing scenario			
SCS trial	£5281	£3441-£7931	Taylor et al^19^ inflated to 2016
Failed SCS trial (electrode removal)	£2140	£921-£3593	Taylor et al^19^ inflated to 2016
Permanent SCS implantation			
10 kHz-SCS	£16,648	£13,116-£21,421*	Annemans et al^14^ inflated to 2016
NRLF-SCS	£11,281	£8888-£14,516	Taylor et al^19^ inflated to 2016
RLF-SCS	£17,422	£13,726-22,418*	Taylor et al^19^ inflated to 2016
SCS explanation	£2140	£0-£3015	Taylor et al^19^ inflated to 2016
SCS-related complication	£740	£241-£1869	Taylor et al^19^ inflated to 2016
Drug pain therapy—CMM alone (6 mo)	£3167	£0-£8412	Taylor et al^19^ inflated to 2016
Non-drug pain therapy—CMM alone (6 mo)	£956	£0-£1157	Taylor et al^19^ inflated to 2016
Drug pain therapy—SCS+CMM (6 mo)	£2012	£0-£8412	Taylor et al^19^ inflated to 2016
Non-drug pain therapy—SCS+CMM	£33	£0-£40	Taylor et al^19^ inflated to 2016
Alternative system costing scenario			
Permanent SCS implantation			
10 kHz-SCS	£16,648*	NR	Taylor et al^19^ inflated to 2016
NRLF-SCS	£11,281	NR	Taylor et al^19^ inflated to 2016
RLF-SCS	£16,648	NR	Conservatively assumed to be equal to 10 kHz-SCS
SCS reimplantation			
10 kHz-SCS	£14,201	NR	Annemans et al^14^ inflated to 2016.* Proportionally reduced to reflect the cost differential between initial and replacement systems for RLF-SCS reported in Taylor et al^19^ inflated to 2016
NRLF-SCS	£10,499	NR	Taylor et al^19^ inflated to 2016
RLF-SCS	£14,201	NR	Conservatively assumed to be equal to 10 kHz-SCS therapy

*No CI data available therefore this analysis assumes the same proportional difference as reported for NRLF-SCS as reported by Taylor et al.^19^

10 kHz-SCS indicates 10 kHz high-frequency spinal cord stimulation; CI, confidence interval; CMM, conventional medical management; NR, not reported; NRLF-SCS, traditional low-frequency nonrechargeable spinal cord stimulation; RLF-SCS, traditional low-frequency rechargeable spinal cord stimulation.

In the base-case, the cost of SCS reimplantation for all devices was assumed to be the same as the respective permanent implantation cost.

For health state costs, the analysis conservatively assumed that the cost of CMM was the same, irrespective of the pain response achieved. The health state costs used in the cost model and costs for AEs not resulting in a device explant included in the analysis are outlined in Table 2.

### Data Analysis and Sensitivity Analysis

#### Cost-consequence Analysis

Deterministic and probabilistic sensitivity analyses (PSA) were conducted to assess the uncertainty surrounding the model inputs and sensitivity of the model results to changes in efficacy and cost. One-way sensitivity analyses were performed using realistic minimum and maximum individual model inputs; for all model parameters, the minimum and maximum plausible values for univariate analysis were defined as the lower and upper 95% confidence limits (95% confidence intervals [CIs]).

All clinical probabilities (eg, SCS trial success, proportion of patients achieving optimal pain relief) were varied using a beta distribution, and all costs and device longevity were varied using a gamma distribution, in line with best practice.^8^,^24^–^27^ Results of univariate sensitivity analyses were depicted on Tornado diagrams, demonstrating how changes in individual model inputs between plausible minimum and maximum values influenced the model results. A Tornado diagram plots the results of the 10 most influential parameters on the outcome (cost) from a sensitivity analysis exercise; however, it should be noted that all parameters were tested. Threshold analysis was also performed on the 10 key model parameters, as identified in the univariate sensitivity analysis, to determine at which values 10 kHz-SCS would be cost-neutral compared with NRLF-SCS and RLF-SCS therapy. All parameters were simultaneously varied in PSA and the results reported as the probability of 10 kHz-SCS being cost-saving based on 5000 simulations.

#### Cost-effectiveness (Cost-utility) Analysis

All results were reported as the ICER (Table 3). Results of PSAs were depicted on scatter plots on the cost-effectiveness plane, showing the distribution of ICERs generated from 5000 simulations. In addition, cost-effectiveness acceptability curves depicted PSA results and demonstrated the probability of 10 kHz-SCS therapy being cost-effective versus NRLF-SCS and RLF-SCS therapy over a range of monetary values that a decision-maker may be willing to pay per QALY.^27^

**TABLE 3:** Base-case Costing and Cost-effectiveness (Cost-utility) Analysis Results

	Costing Analysis*		Cost-effectiveness Analysis		
Treatment	Total Costs	Δ Costs Versus 10 kHz-SCS	Total QALYs	Δ QALYs Versus 10 kHz-SCS	ICER Versus 10 kHz-SCS
10 kHz-SCS	£87,400	—	5.268	—	—
NRLF-SCS	£95,156	£7755	4.352	−0.916	NRLF-SCS dominated† by 10 kHz-SCS
RLF-SCS	£92,196	£4795	4.355	−0.913	RLF-SCS dominated† by 10 kHz-SCS

*Total costs/incremental costs from costing analysis feed into the cost-effectiveness analysis.

†Dominated=higher costs and lower QALYs.

10 kHz-SCS indicates 10 kHz high-frequency spinal cord stimulation; CMM, conventional medical management; ICER, incremental cost-effectiveness ratio; NRLF-SCS, traditional low-frequency non-rechargeable spinal cord stimulation; RLF-SCS, traditional low-frequency rechargeable spinal cord stimulation; QALYs, quality-adjusted life years.

## RESULTS

### Cost-consequence Analysis

#### Base-case Analysis

The base-case analysis shows that 10 kHz-SCS would have a total mean cost of £87,400 per patient over 15 years compared with £95,156 for NRLF-SCS and £92,196 for RLF-SCS. In both instances 10 kHz-SCS is cost-saving compared with NRLF-SCS and RLF-SCS (Table 3).

A summary of costs by category of cost per patient for 10 kHz-SCS versus NRLF-SCS and RLF-SCS is shown in the e-appendix (e-Tables 1 and 2, Supplemental Digital Content 1, http://links.lww.com/CJP/A666, Supplemental Digital Content 2, http://links.lww.com/CJP/A667, respectively). It should be noted that costs recorded for 10 kHz-SCS are in part higher initially because there are more trial successes in that group and therefore more patients go ahead to full implant as a result of the increased efficacy versus both NRLF-SCS and RLF-SCS.

A summary of costs by health state is also provided for 10 kHz-SCS versus NRLF SCS and RLF-SCS in the e-appendix (e-Tables 3 and 4, Supplemental Digital Content 3, http://links.lww.com/CJP/A668, Supplemental Digital Content 4, http://links.lww.com/CJP/A669, respectively).

A summary of costs by AEs per patient showed that device-related complications accounted for all AE costs. 10 kHz-SCS was associated with lower device-related AE costs compared with both NRLF-SCS and RLF-SCS (10 kHz-SCS vs. NRLF-SCS: £387 vs. £712; 10 kHz-SCS vs. RLF-SCS: £387 vs. £600).

#### Sensitivity Analyses

Within univariate analysis very few scenarios resulted in greater incremental costs for 10 kHz-SCS. The PSA shows the results to be very stable with >70% of simulations resulting in cost-savings versus both NRLF-SCS and RLF-SCS.

**FIGURE 2:**
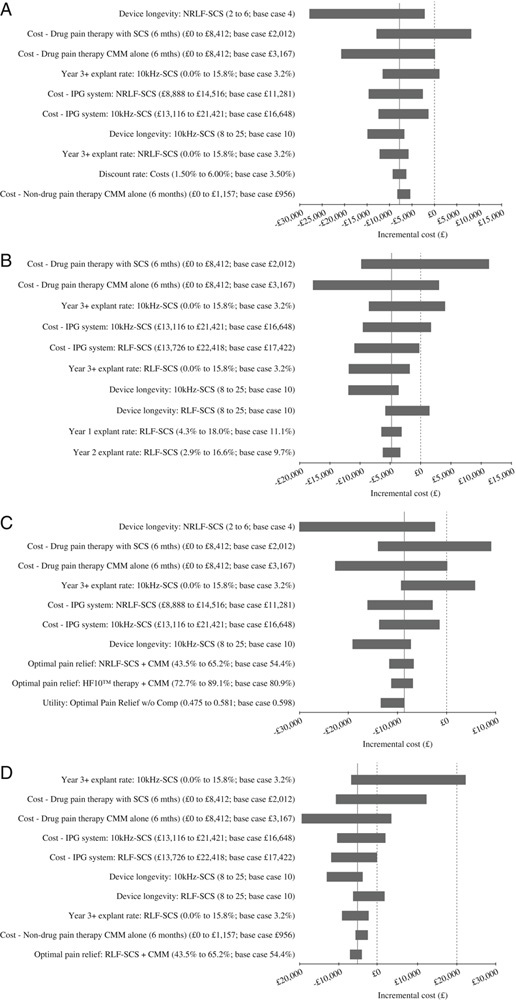
Tornado diagrams for 10 kHz-SCS versus NRLF-SCS and RLF-SCS cost-consequence (A, B) and cost-utility analysis (C, D). A and B, The solid vertical line represents the base case cost for 10 kHz-SCS versus NRLF-SCS and RLF SCS, respectively. A negative figure on the *x*-axis indicates that 10 kHz SCS is cost-saving. The dash vertical line represents the cost at which 10 kHz SCS is cost-neutral (ie, £0). C and D, The solid vertical line represents the base-case ICER and a negative figure on the *x*-axis indicates that 10 kHz-SCS is the dominant treatment strategy. The dash vertical line represents the ICER at which 10 kHz-SCS is dominant. The dot-dash vertical line represents the WTP threshold in the United Kingdom (£20,000/QALY). 10 kHz-SCS indicates 10 kHz high-frequency spinal cord stimulation; CMM, conventional medical management; IPG, interventional procedure guidance; NRLF-SCS/TNR-SCS, traditional low-frequency nonrechargeable spinal cord stimulation; RLF-SCS/TR-SCS, traditional low-frequency rechargeable spinal cord stimulation.

One-way (univariate) sensitivity analyses were performed to assess the robustness of the results generated by the model. Key parameters were individually varied across a plausible range of values. The results of the sensitivity analyses, comparing 10 kHz-SCS with NRLF-SCS and RLF-SCS, are represented in tornado diagrams in Figures 2A and B, respectively, where the solid vertical line represents the base-case incremental cost.

Threshold analysis was performed confirming that 10 kHz-SCS was cost-saving compared with NRLF-SCS and RLF-SCS therapy. Specific criteria required for 10 kHz-SCS to be cost-neutral compared with both therapies are presented in the e-appendix (e-appendix, Threshold analysis, e-Tables 5 and 6, Supplemental Digital Content 5, http://links.lww.com/CJP/A670, Supplemental Digital Content 6, http://links.lww.com/CJP/A671).

Multiway sensitivity analyses were performed to assess the impact of using back pain relief values taken from SENZA-RCT, (assessed at 3, 12, and 24 mo), time horizon, and alternative system costs on the incremental cost of 10 kHz-SCS versus NRLF-SCS and RLF-SCS. This shows that 10 kHz-SCS remains cost-saving irrespective of these model parameters (e-appendix, Multiway sensitivity analyses, e-Tables 7–11, Supplemental Digital Content 7, http://links.lww.com/CJP/A672, Supplemental Digital Content 8, http://links.lww.com/CJP/A673, Supplemental Digital Content 9, http://links.lww.com/CJP/A674, Supplemental Digital Content 10, http://links.lww.com/CJP/A675, Supplemental Digital Content 11, http://links.lww.com/CJP/A676, e-Fig. 1, Supplemental Digital Content 12, http://links.lww.com/CJP/A677).

The results of the PSA were robust with 10 kHz-SCS remaining cost-saving in 74% of simulations performed compared with NRLF-SCS therapy and 73% compared with RLF-SCS therapy. The mean cost-saving is £7170 per patient (95% CI: £6767-£7573) versus NRLF-SCS therapy and £3552 per patient (95% CI: £3313-£3792) versus RLF-SCS therapy.

### Cost-effectiveness (Cost-utility) Analysis

#### Base-case Analysis

Our base-case cost-effectiveness analysis shows 10 kHz-SCS is cost-saving and results in more QALYs than NRLF-SCS and RLF-SCS, tha is, it is economically dominant (Table 3).

#### Sensitivity Analysis

One-way (univariate) sensitivity analyses were performed to assess the robustness of the cost-effectiveness results utilizing the previously defined ranges. The results of the sensitivity analyses, comparing 10 kHz-SCS with NRLF-SCS and RLF-SCS, are represented in Tornado diagrams in Figures 2C and D, respectively. The key drivers are broadly aligned with those in the cost-consequence analysis and only the explant rate beyond Year 3 for 10 kHz-SCS, at the highest value (15.8%), resulted in an ICER above the WTP threshold of £20,000 per QALY for 10 kHz-SCS compared with RLF-SCS.

The threshold analysis on the 10 key drivers demonstrates that the parameter values required for 10 kHz-SCS to be cost-effective at a WTP threshold of £20,000 per QALY are pushed out further than the original cost-consequence analysis.

**FIGURE 3:**
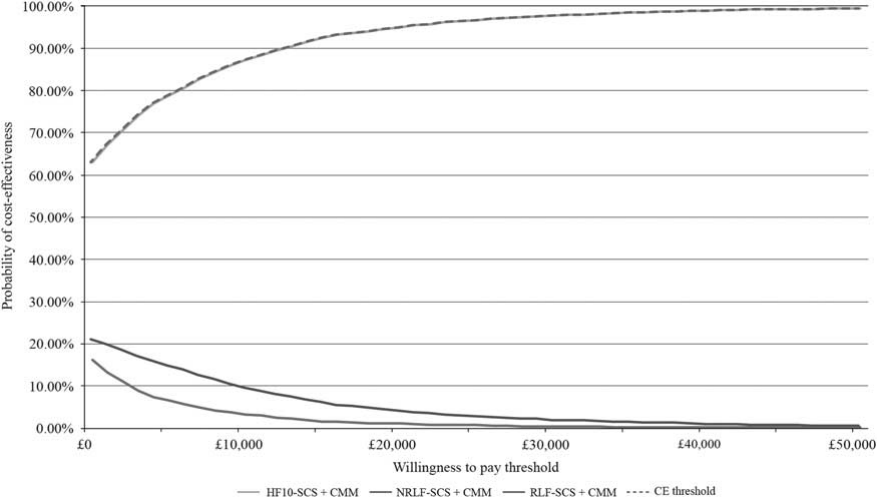
Cost-effectiveness acceptability curves for 10 kHz-SCS therapy versus NRLF-SCS and RLF-SCS. 10 kHz-SCS/HF-10-SCS indicates 10 kHz high-frequency spinal cord stimulation; CMM, conventional medical management; NRLF-SCS/TNR-SCS, traditional low-frequency nonrechargeable spinal cord stimulation; RLF-SCS/TR-SCS, traditional low-frequency rechargeable spinal cord stimulation.

The cost-effectiveness acceptability curve for the base-case scenario shows that 10 kHz-SCS therapy has a 95% likelihood of being cost-effective at a WTP threshold of £20,000 per QALY (Fig. 3).

## DISCUSSION

The findings of this study show that 10 kHz-SCS is both cost-saving and cost-effective compared with LF-SCS, across a wide range of sensitivity analyses. These data are consistent with those published in the NICE 2019 Medical Technologies Guidance (MTG41)^15^ and lend additional support to the case for choosing 10 kHz-SCS over LF-SCS, with either rechargeable or nonrechargeable batteries, for patients with CBLP.

### Strengths and Limitations

Our analysis has several strengths. First, the SENZA-RCT provided head-to-head data on pain relief from a direct comparison of patients treated either by 10 kHz-SCS or by LF-SCS for CBLP (and some with FBSS).^9^,^10^ Second, our analysis was based on an established cost-effectiveness model for SCS, with assumptions that have been previously critiqued and accepted by NICE.^8^,^16^ We used explant rates derived from the SENZA-RCT, in which patients were randomized to receive either 10 kHz-SCS or LF-SCS devices and followed up in a controlled trial for 24 months. In our analysis, the LF-SCS group had higher explant rates compared with 10 kHz-SCS. Although, reasons for explantation was not reported here, previous studies have identified inadequate pain relief, loss of efficacy and uncomfortable paresthesia as key factors.^28^–^31^ LF-SCS had lower pain relief and response rate and had higher rate of uncomfortable parsthesia compared with 10 kHz SCS in SENZA-RCT.^9^,^10^ In contrast, the apparently higher explant rates reported by Van Buyten et al^32^ in their retrospective chart review over a median observation period of 2.24 years (circa 27 mo) are subject to reporting bias due to the exclusion of 52.4% of the explants occurring as a result of battery depletion of non-rechargeable devices. The Van Buyten analysis was at risk of selection bias due to reimbursement eligibility in Belgium that limits the selection of rechargeable SCS (10 kHz-SCS and RLF-SCS) to only the most severe and clinically complex patients. These considerations explain why the outcomes from the Van Buyten study differ from ours and why it was not used in preference to patient level data from SENZA-RCT to inform explant rate parameters in our analysis. In a scenario analysis, assuming an equivalent rate of device explant of 3.2% each year for all device types, although the incremental cost and QALY differences are reduced, 10 kHz-SCS remains cost-saving and results in more QALYs than NRLF-SCS and RLF-SCS and thus remains a dominant treatment strategy. Third, our 15-year time horizon allowed consideration of long-term costs and outcomes, including the cost and clinical impact of SCS-related complications, and device explant and replacement. It could be argued that comparatively higher explant rates and nonserious complications in LF-SCS group contributed to higher costs Lead migration rate, which was reported to be slightly but not significantly (*P*=0.49) higher in the LF-SCS group can also be hypothesized to contribute to the higher costs of LF-SCS.^9^,^10^,^14^ In a another scenario analysis, assuming equal rates of nonserious complications for all devices types 10 kHz-SCS remains the dominant treatment strategy. This mirrors the results of the previous cost-efficiency analysis by Annemans and colleagues, which assumed similar complication rate and withdrawal rate between 10 kHz SCS and LF-SCS groups and also showed the dominance of 10 kHz-SCS. Finally, we undertook extensive sensitivity analyses that comprehensively reflected uncertainty in model inputs and assumptions, which showed our findings to be robust.

A potential limitation of our analysis is the lack of real-world data on the use of SCS beyond 2 years, and therefore longer-term device and battery longevity remain uncertain. This is particularly important for nonrechargeable LF-SCS which are expected to have a much shorter service life and therefore require replacement at more frequent intervals. However, our sensitivity analysis showed that the longevity of the 10 kHz-SCS device could be 4-years lower than the anticipated longevity of 10-years and remain cost-saving and cost-effective at a £20,000/QALY WTP threshold. An important further consideration is that our economic modelling did not recognize the potential value of a magnitude of pain relief beyond the 50% reduction in pain measured on a VAS. The “remitter” state of achievement of an absolute level of pain VAS of <3.0 cm as a result of 10 kHz-SCS has been associated with significant functional improvement and is likely to be associated with additional HRQoL gain.^33^ In addition, our modelling did not address 3 further important advantages of 10 kHz-SCS over LF-SCS. First, no paresthesia means 10 kHz-SCS treatment can be continued during sleep and while driving or operating machinery, improving the continuity of pain reduction and so improving HRQoL. Some patients may continually use their SCS device 24 hours per day while others may continually use the device for only a few hours per day. Paresthesia sensation is likely to be a factor in usage patterns for traditional LF-SCS. From the SENZA-RCT, patients used 10 kHz-SCS therapy for an average of 24 (SD 0.1) hours per day and LF-SCS for an average of 17.0 (SD 7.3) hours per day (data obtained from manufacturer). A survey of 2955 US patients using 10 kHz-SCS reported that 99% were able to sleep and 99% drive a vehicle with the stimulation switched on.^22^ Second, no need for paresthesia mapping during implantation of 10 kHz-SCS means shorter and more predictable procedure times,^20^ so potentially reducing implantation costs.^34^ Third, superior pain relief with 10 kHz-SCS is likely to reduce concomitant opioid medication.^20^,^35^,^36^

### Implications for Policy and Practice

Our finding that 10 kHz-SCS is cost-saving relative to LF-SCS lends further support to the NICE Medical Technologies Guidance recommendation.^15^ NICE 2019 guidance is based on technologies being cost-saving (on analysis of cost-consequences) and at least as effective as the comparator; or else cost-neutral with additional benefits or effectiveness.^27^ It should be noted that our base-case analysis used an acquisition cost for LF nonrechargeable SCS devices of £11,281 (some two thirds of the base-case cost for a LF rechargeable SCS device and 10 kHz-SCS device). This price differential is reasonable in Europe but not generalizable to all jurisdictions, particularly the United States where rechargeable and nonrechargeable devices are reimbursed at the same rate. Consequently, the reported cost-savings for 10 kHz-SCS and rechargeable LF-SCS would be expected to be much greater if considered in the US context, given the similarity in reimbursement rates and reduced service life of nonrechargeable devices.

A further important policy consideration is the potential reduction in concomitant opioid analgesia usage in patients receiving 10 kHz-SCS.^20^,^35^ Recent studies have shown that 10 kHz-SCS is an opioid-sparing treatment that significantly reduces the overall dose of opioids in individuals with CBLP and reduces the proportion of individuals requiring high-risk doses >90 MME per day.^20^,^35^ SENZA-RCT has shown these reductions in mean morphine equivalent dose to be greater with 10 kHz-SCS compared with LF-SCS (18.8% average reduction at 12 months compared with 1% for LF-SCS) which is a statistically significant difference.^9^

Our full economic evaluation (cost-effectiveness analysis) demonstrates that 10 kHz-SCS results in more QALYs or patient health gain (economic dominance), that is, it is cost-effective in addition to providing cost-consequence benefits. This combination of findings provides a strong economic case for patients and health care providers to choose 10 kHz-SCS over traditional LF-SCS devices (either rechargeable or nonrechargeable). The additional advantages of no paresthesia sensation and no need for paresthesia mapping during implantation, provide further justification for patients and providers of health care to prefer 10 kHz-SCS over other SCS devices.

### Implications for Future Research

With the development of a plethora of SCS waveforms and continued incremental development in technology (including MRI considerations), rigorous economic analyses are needed to ensure the value for money of these innovations. During this analysis, we identified 2 areas where future research would be particularly beneficial. First, further long-term, real-world data, would be useful to provide reliable and precise information about the longevity of SCS devices, particularly those with non-rechargeable batteries. Secondly, more information is needed about patients who become “remitters” following implantation. The SENZA-RCT and other published studies of 10 kHz-SCS have demonstrated that a substantial proportion of patients experience sustained pain relief considerably greater than 50%, with sustained pain scores of ≤3.0 (on a 0 to 10 VAS)—so-called “remitter” state.^9^,^10^,^20^,^33^,^35^,^37^–^42^ This contrasts with the assumption in the model that treatment had been successful if patients achieved ≥50%: the real-world outcomes with use of 10 kHz-SCS are better than this.^38^ Research into the HRQoL and health care costs of remitters would enable an update of the existing SCS economic model.

## CONCLUSIONS

This analysis has shown that 10 kHz-SCS is both cost-saving and cost-effective compared with LF-SCS, for patients with CBLP including those with FBSS, with an ICER well below a WTP threshold of £20,000/QALY. The magnitude of cost-saving is greater when 10 kHz-SCS is compared against nonrechargeable SCS devices. These findings are consistent with the recently published NICE Medical Technologies Guidance (MTG41) recommendation for health care payers, clinicians and patients,^15^ and provide further support for the economic case to choose 10 kHz-SCS over LF-SCS. 10 kHz-SCS has additional advantages, not formally captured in our analysis, relating to shorter and more predictable procedure times; and consequent health-related quality of life benefits for patients.

## Supplementary Material

SUPPLEMENTARY MATERIAL

Supplemental Digital Content is available for this article. Direct URL citations appear in the printed text and are provided in the HTML and PDF versions of this article on the journal's website, www.clinicalpain.com.

## References

[1] Vos T Allen C Arora M Global, regional, and national incidence, prevalence, and years lived with disability for 310 diseases and injuries, 1990–2015: a systematic analysis for the Global Burden of Disease Study 2015. Lancet. 2016;388:1545–1602. 27733282 10.1016/S0140-6736(16)31678-6 PMC5055577

[2] Maniadakis N Gray A The economic burden of back pain in the UK. Pain. 2000;84:95–103. 10601677 10.1016/S0304-3959(99)00187-6

[3] Martin BI Deyo RA Mirza SK Expenditures and health status among adults with back and neck problems. JAMA. 2008;299:656–664. 18270354 10.1001/jama.299.6.656

[4] Kapural L Peterson E Provenzano DA Clinical evidence for spinal cord stimulation for failed back surgery syndrome (FBSS). Spine. 2017;42:S61–S66. 28441313 10.1097/BRS.0000000000002213

[5] Mekhail N Visnjevac O Azer G Spinal cord stimulation 50 years later: clinical outcomes of spinal cord stimulation based on randomized clinical trials—a systematic review. Reg Anesth Pain Med. 2018;43:391–406. 29481371 10.1097/AAP.0000000000000744

[6] Cruccu G Garcia-Larrea L Hansson P EAN guidelines on central neurostimulation therapy in chronic pain conditions. Eur J Neurol. 2016;23:1489–1499. 27511815 10.1111/ene.13103

[7] Manchikanti L Abdi S Atluri S An update of comprehensive evidence-based guidelines for interventional techniques in chronic spinal pain. Part II: guidance and recommendations. Pain Physician. 2013;16:S49–S283. 23615883

[8] National Institute for Health and Care Excellence. Spinal cord stimulation for chronic pain of neuropathic or ischaemic origin. Technology appraisal guidance (TA159). 2008. Available at: https://www.nice.org.uk/guidance/ta159. Accessed August 2019.

[9] Kapural L Yu C Doust MW Novel 10-kHz high-frequency therapy (HF10 therapy) is superior to traditional low-frequency spinal cord stimulation for the treatment of chronic back and leg pain: the SENZA-RCT randomized controlled trial. Anesthesiology. 2015;123:851–860. 26218762 10.1097/ALN.0000000000000774

[10] Kapural L Yu C Doust MW Comparison of 10-kHz high-frequency and traditional low-frequency spinal cord stimulation for the treatment of chronic back and leg pain: 24-month results from a multicenter, randomized, controlled pivotal trial. Neurosurgery. 2016;79:667–677. 27584814 10.1227/NEU.0000000000001418 PMC5058646

[11] US Food and Drug Administration. Premarket Approval (PMA). Nevro Senza spinal cord stimulation (SCS) system. 2019. Availablet at: https://www.accessdata.fda.gov/scripts/cdrh/cfdocs/cfpma/pma.cfm?id=P130022 Accessed August 2019.

[12] Hoelscher C Riley J Wu C Cost-effectiveness data regarding spinal cord stimulation for low back pain. Spine. 2017;42:S72–S79. 28399549 10.1097/BRS.0000000000002194

[13] Rawlins MD Culyer AJ National Institute for Clinical Excellence and its value judgments. BMJ. 2004;329:224–227. 15271836 10.1136/bmj.329.7459.224 PMC487742

[14] Annemans L Van Buyten J-P Smith T Cost effectiveness of a novel 10 kHz high-frequency spinal cord stimulation system in patients with failed back surgery syndrome (FBSS). J Long Term Eff Med Implants. 2014;24:173–183. 25272216 10.1615/jlongtermeffmedimplants.2014011685

[15] National Institute for Health and Care Excellence. Medical technologies guidance 41 (MTG41). Senza spinal cord stimulation system for delivering HF10 therapy to treat chronic neuropathic pain. 2019. Available at: https://www.nice.org.uk/guidance/mtg41/resources/senza-spinal-cord-stimulation-system-for-delivering-hf10-therapy-to-treat-chronic-neuropathic-pain-pdf-64372050739141. Accessed August 2019.

[16] National Institute for Health and Care Excellence. SENZA™ spinal cord stimulation for the treatment of chronic pain. Medical Technologies Evaluation Programme (MT515). 2017. Available at: https://www.nice.org.uk/guidance/mtg41/documents/supporting-documentation-2 Accessed August 2019.

[17] Husereau D Drummond M Petrou S Consolidated Health Economic Evaluation Reporting Standards (CHEERS) Statement. Cost Eff Resour Alloc. 2013;11:6. 23531194 10.1186/1478-7547-11-6 PMC3607888

[18] National Institute for Health and Care Excellence. Guide to the methods of technology appraisal 2013. Process and methods [PMG9]. 2013. Available at: https://www.nice.org.uk/process/pmg9/chapter/foreword Accessed August 2019. 27905712

[19] Taylor RS Ryan J O’Donnell R The cost-effectiveness of spinal cord stimulation in the treatment of failed back surgery syndrome. Clin J Pain. 2010;26:463–469. 20551721 10.1097/AJP.0b013e3181daccec

[20] DiBenedetto DJ Wawrzyniak KM Schatman ME 10 kHZ spinal cord stimulation: a retrospective analysis of real-world data from a community-based, interdisciplinary pain facility. J Pain Res. 2018;11:2929–2941. 30538532 10.2147/JPR.S188795 PMC6251433

[21] Simpson E Duenas A Holmes M Spinal cord stimulation for chronic pain of neuropathic or ischaemic origin: systematic review and economic evaluation. *NIHR Health Technology Assessment programme: Executive Summaries*. NIHR Journals Library; 2009. 10.3310/hta13170 19331797

[22] Nevro Corporation. Data on File. SENZA-RCT. 2017.

[23] Curtis L and Burns A Unit Costs of Health and Social Care 2016, PSSRU. 2016. Available at: https://www.pssru.ac.uk/project-pages/unit-costs/unit-costs-2016/ Accessed August 2019.

[24] Briggs AH Handling uncertainty in cost-effectiveness models. Pharmacoeconomics. 2000;17:479–500. 10977389 10.2165/00019053-200017050-00006

[25] Briggs AH Goeree R Blackhouse G Probabilistic analysis of cost-effectiveness models: choosing between treatment strategies for gastroesophageal reflux disease. Med Decis Making. 2002;22:290–308. 12150595 10.1177/0272989X0202200408

[26] Fenwick E Claxton K Sculpher M Representing uncertainty: the role of cost-effectiveness acceptability curves. Health Econ. 2001;10:779–787. 11747057 10.1002/hec.635

[27] National Institute for Health and Care Excellence. Medical technologies evaluation programme methods guide. Process and methods [PMG33]. 2017. Available at: https://www.nice.org.uk/process/pmg33/chapter/introduction Accessed August 2019. 27905707

[28] Simopoulos T Aner M Sharma S Explantation of percutaneous spinal cord stimulator devices: a retrospective descriptive analysis of a single-center 15-year experience. Pain Med. 2019;20:1355–1361. 30889248 10.1093/pm/pny245

[29] Patel SK Gozal YM Saleh MS Spinal cord stimulation failure: evaluation of factors underlying hardware explantation. J Neurosurg Spine. 2019:1–6. 10.3171/2019.6.SPINE181099 31585414

[30] Pope JE Deer TR Falowski S Multicenter retrospective study of neurostimulation with exit of therapy by explant. Neuromodulation. 2017;20:543–552. 28714533 10.1111/ner.12634

[31] Dupre DA Tomycz N Whiting D Spinal cord stimulator explantation: motives for removal of surgically placed paddle systems. Pain Pract. 2018;18:500–504. 28875558 10.1111/papr.12639

[32] Van Buyten JP Wille F Smet I Therapy-related explants after spinal cord stimulation: results of an international retrospective chart review study. Neuromodulation. 2017;20:642–649. 28834092 10.1111/ner.12642 PMC5656934

[33] Amirdelfan K Gliner BE Kapural L A proposed definition of remission from chronic pain, based on retrospective evaluation of 24-month outcomes with spinal cord stimulation. Postgrad Med. 2019;131:278–286. 30901266 10.1080/00325481.2019.1592401

[34] Bourke C Baranidaran G Bush D Can HF10 implantation time be reliably planned? The experience of Leeds Teaching Hospital Trust in performing 10 full implants on one day. International Neuromodulation Society; 2017.

[35] Al-Kaisy A Van Buyten JP Carganillo R 10 kHz SCS therapy for chronic pain, effects on opioid usage: post hoc analysis of data from two prospective studies. Sci Rep. 2019;9:11441. 31391503 10.1038/s41598-019-47792-3 PMC6686020

[36] Al-Kaisy A Van Buyten JP Amirdelfan K Opioid-sparing effects of 10 kHz spinal cord stimulation: a review of clinical evidence. Ann N Y Acad Sci. 2020;1462:53–64. 31578744 10.1111/nyas.14236 PMC7065058

[37] Salmon J High-frequency spinal cord stimulation at 10 kHz for widespread pain: a retrospective survey of outcomes from combined cervical and thoracic electrode placements. Postgrad Med. 2019;131:230–238. 30807247 10.1080/00325481.2019.1587564

[38] Stauss T El Majdoub F Sayed D A multicenter real-world review of 10 kHz SCS outcomes for treatment of chronic trunk and/or limb pain. Ann Clin Transl Neurol. 2019;6:496–507. 30911573 10.1002/acn3.720 PMC6414485

[39] Amirdelfan K Vallejo R Benyamin R High-frequency spinal cord stimulation at 10 kHz for the treatment of combined neck and arm pain: results from a prospective multicenter study. Neurosurgery. 2020;87:176–185. 31792530 10.1093/neuros/nyz495 PMC7360873

[40] Al-Kaisy A Van Buyten JP Kapural L 10 kHz spinal cord stimulation for the treatment of non-surgical refractory back pain: subanalysis of pooled data from two prospective studies. Anaesthesia. 2020;75:775–784. 32383509 10.1111/anae.15036 PMC7384077

[41] Kapural L Gupta M Paicius R Treatment of chronic abdominal pain with 10 kHz spinal cord stimulation: safety and efficacy results from a 12-month prospective, multicenter, feasibility study. Clin Transl Gastroenterol. 2020;11:e00133. 32463618 10.14309/ctg.0000000000000133 PMC7145032

[42] Sayed D Kallewaard JW Rotte A Pain relief and improvement in quality of life with 10 kHz SCS therapy: summary of clinical evidence. CNS Neurosci Ther. 2020;26:403–415. 32087613 10.1111/cns.13285 PMC7080433

