# Supplementary material for: High-frequency 10 kHz Spinal Cord Stimulation for Chronic Back and Leg Pain: Cost-consequence and Cost-effectiveness Analyses
Source: Clin J Pain. 2020 Aug 4;36(11):852–61. doi: 10.1097/AJP.0000000000000866 (PMC7671822; doi:10.1097/AJP.0000000000000866)
Supplement: SUPPLEMENTARY MATERIAL [file ajp-36-852-s001.docx]

# SUPPLEMENTARY MATERIALS

## Costs per patient

**e-Table 1 - Summary of costs by category of cost per patient: 10kHz‑SCS versus NRLF‑SCS**

| **Cost category** | **10kHz‑SCS therapy** | **NRLF‑SCS therapy** | **Cost increment** | **Absolute increment (cost)** | **Absolute increment (%)** |
| --- | --- | --- | --- | --- | --- |
| Initial trial | £5,281 | £5,281 | £0 | £0 | 0% |
| Permanent implant (successful trial) | £15,449 | £9,928 | £5,522 | £5,522 | 29% |
| Explant (failed trial) | £154 | £257 | -£103 | £103 | 1% |
| Pain management and complication costs | £58,150 | £63,588 | -£5,439 | £5,439 | 29% |
| Reimplantation | £7,120 | £14,479 | -£7,359 | £7,359 | 39% |
| Explant | £624 | £739 | -£114 | £114 | 1% |
| Revision | £621 | £884 | -£262 | £262 | 1% |
| **Total** | **£87,400** | **£95,156** | **-£7,755** | **£18,799** | **100%** |

Abbreviations: 10kHz‑SCS, 10kHz high frequency spinal cord stimulation; NRLF‑SCS, non‑rechargeable low-frequency spinal cord stimulation.
