# Supplementary material for: High-frequency 10 kHz Spinal Cord Stimulation for Chronic Back and Leg Pain: Cost-consequence and Cost-effectiveness Analyses
Source: Clin J Pain. 2020 Aug 4;36(11):852–61. doi: 10.1097/AJP.0000000000000866 (PMC7671822; doi:10.1097/AJP.0000000000000866)
Supplement: SUPPLEMENTARY MATERIAL [file ajp-36-852-s003.docx]

## Costs by health state

**e-Table 3 - Summary of costs by health state per patient: 10kHz-SCS versus NRLF-SCS**

| **Health state** | **10kHz‑SCS therapy** | **NRLF‑SCS therapy** | **Cost increment** | **Absolute increment (cost)** | **Absolute increment (%)** |
| --- | --- | --- | --- | --- | --- |
| Optimal pain relief without complications | £29,588^†^ | £19,176 | £10,411 | £10,411 | 40% |
| Optimal pain relief with complications | £1,180 | £1,457 | -£278 | £278 | 1% |
| Sub‑optimal pain relief without complications | £27,103 | £41,733 | -£14,629 | £14,629 | 56% |
| Sub‑optimal pain relief with complications | £279 | £1,222 | -£943 | £943 | 4% |
| **Total** | **£58,150** | **£63,588** | **-£5,439** | **£26,262** | **100%** |

Abbreviations: 10kHz‑SCS, 10kHz high frequency spinal cord stimulation; NRLF‑SCS, non‑rechargeable low-frequency spinal cord stimulation; RCT, randomised controlled trial.

† This cost is initially higher for 10kHz‑SCS therapy as more patients achieve optimal pain relief and go on to full implant versus NRLF-SCS and RLF-SCS (higher responder rate from SENZA‑RCT).
