# Supplementary material for: High-frequency 10 kHz Spinal Cord Stimulation for Chronic Back and Leg Pain: Cost-consequence and Cost-effectiveness Analyses
Source: Clin J Pain. 2020 Aug 4;36(11):852–61. doi: 10.1097/AJP.0000000000000866 (PMC7671822; doi:10.1097/AJP.0000000000000866)
Supplement: SUPPLEMENTARY MATERIAL [file ajp-36-852-s004.docx]

**e-Table 4 - Summary of costs by health state per patient: 10kHz‑SCS versus RLF-SCS**

| **Health state** | **10kHz‑SCS therapy** | **RLF‑SCS therapy** | **Cost increment** | **Absolute increment (cost)** | **Absolute increment (%)** |
| --- | --- | --- | --- | --- | --- |
| Optimal pain relief without complications | £29,588^†^ | £19,343 | £10,244 | £10,244 | 40% |
| Optimal pain relief with complications | £1,180 | £1,230 | -£50 | £50 | 0% |
| Sub optimal pain relief without complications | £27,103 | £41,873 | -£14,769 | £14,769 | 57% |
| Sub optimal pain relief with complications | £279 | £1,031 | -£752 | £752 | 3% |
| **Total** | **£58,150** | **£63,477** | **-£5,328** | **£25,817** | **100%** |

Abbreviations: 10kHz‑SCS, 10kHz high frequency spinal cord stimulation; RCT, randomised controlled trial; RLF-SCS, rechargeable low-frequency spinal cord stimulation.

† This cost is initially higher for 10kHz‑SCS therapy as more patients achieve optimal pain relief and go on to full implant versus NRLF-SCS and RLF-SCS (higher responder rate from SENZA‑RCT).

Threshold analysis

In the threshold analysis (i.e. when parameters are considered individually) for 10kHz high frequency spinal cord stimulation (10kHz‑SCS) to be cost‑neutral compared with non‑rechargeable low-frequency spinal cord stimulation (NRLF-SCS):

- NRLF-SCS system device longevity is >7.5 years, which is outside the plausible range presented
- The cost of the drug pain therapy element of conventional medical management (CMM) given with spinal cord stimulation (SCS) would need to be more than double the cost (£5,097)
- The explant rate for the 10kHz‑SCS would need to be 13.6% in year 3 onwards
- The following costs would need to be negative which is impossible:
  - Drug pain therapy element of CMM given alone
  - Explant rate for the NRLF-SCS system in Year 3 onwards
- The cost of the 10kHz‑SCS would need to increase to £22,368, or the cost of the NRLF‑SCS system would need to drop to £7,697 (which is below the lower defined confidence interval [CI])

No device longevity figures could be identified for 10kHz‑SCS or NRLF-SCS that would result in cost-neutrality.

Similarly, for 10kHz‑SCS to be cost‑neutral compared with rechargeable low-frequency spinal cord stimulation (RLF-SCS):

- The cost of the drug pain therapy element of CMM given with SCS would need to increase to £3,919
- The annual explant rate for 10kHz‑SCS for Year 3 onwards would need to more than double to 8.8%
- The cost of the 10kHz‑SCS implantation would need to increase to £20,185 or the cost of the RLF-SCS device implantation would need to drop to £13,547
- The explant rate for the RLF-SCS system would need to be negative in Years 1, 2 and 3 onwards (which is not possible)

The device longevity for 10kHz‑SCS would need to drop below 6.75 years which is lower than the defined plausible range, or RLF-SCS longevity would need to increase above 15.25 years.
