# Supplementary material for: High-frequency 10 kHz Spinal Cord Stimulation for Chronic Back and Leg Pain: Cost-consequence and Cost-effectiveness Analyses
Source: Clin J Pain. 2020 Aug 4;36(11):852–61. doi: 10.1097/AJP.0000000000000866 (PMC7671822; doi:10.1097/AJP.0000000000000866)
Supplement: SUPPLEMENTARY MATERIAL [file ajp-36-852-s005.docx]

**e-Table 5 - Results of threshold analysis for 10kHz‑SCS versus NRLF-SCS**

| **Variable** | **Base‑case (CI: lower – upper or range)** | **Cost-consequence analysis:**  **Cost‑neutral value** | **Cost-effectiveness analysis:**  **Value to achieve ICER of £20,000/QALY** |
| --- | --- | --- | --- |
| Device longevity: NRLF-SCS | 4 (2 to 6) | 7.5^‡^ | NA |
| Cost - Drug pain therapy with SCS  (6 months) | £2,012  (£0 to £8,412) | £5,097 | £12,383 |
| Cost - Drug pain therapy CMM alone (6 months) | £3,167  (£0 to £8,412) | £49 | -£7,316^†^ |
| Year 3+ explant rate: 10kHz‑SCS | 3.2%  (0.0% to 15.8%) | 13.6% | 18.1% |
| Cost - IPG system: NRLF-SCS | £11,281  (£8,888 to £14,516) | £7,697 | -£770^†^ |
| Cost - IPG system: 10kHz‑SCS | £16,648  (£13,116 to £21,421) | £22,368 | £35,880 |
| Device longevity: 10kHz‑SCS | 10 (8 to 25) | NA | NA |
| Year 3+ explant rate: NRLF-SCS | 3.2%  (0.0% to 15.8%) | -5.9%^†^ | Not a top 10 driver |
| Discount rate: Costs | 3.50%  (1.50% to 6.00%) | 26.04% | Not a top 10 driver |
| Cost - Non-drug pain therapy CMM alone (6 months) | £956 (£0 to £1,157) | -£2,162^†^ | Not a top 10 driver |
| Optimal pain relief: NRLF-SCS | 54.4%  (43.5% to 65.2%) | Not a top 10 driver | NA |
| Optimal pain relief: 10kHz‑SCS | 80.9%  (72.7% to 89.1%) | Not a top 10 driver | NA |
| Utility: Optimal pain relief without complication | 0.598  (0.475 to 0.581) | Not a top 10 driver | NA |

Abbreviations: 10kHz‑SCS, 10kHz high-frequency spinal cord stimulation; CI, confidence interval; CMM, conventional medical management; ICER, incremental cost‑effectiveness ratio; IPG, interventional procedure guidance; NA, not applicable; NRLF-SCS, non‑rechargeable low‑frequency non‑rechargeable spinal cord stimulation; QALY, quality‑adjusted life year; SCS, spinal cord stimulation.

† These values are not the values for cost-neutrality. Due to the quarterly cycle length it is not possible to have a zero‑cost difference. The figures reported indicate the point at which 10kHz‑SCS is more costly.

‡ These values are negative and would not occur in reality, i.e. they are outside a plausible range.
