# Supplementary material for: High-frequency 10 kHz Spinal Cord Stimulation for Chronic Back and Leg Pain: Cost-consequence and Cost-effectiveness Analyses
Source: Clin J Pain. 2020 Aug 4;36(11):852–61. doi: 10.1097/AJP.0000000000000866 (PMC7671822; doi:10.1097/AJP.0000000000000866)
Supplement: SUPPLEMENTARY MATERIAL [file ajp-36-852-s006.docx]

**e-Table 6 - Results of threshold analysis for 10kHz‑SCS versus RLF-SCS**

| **Variable** | **Base‑case (CI: lower – upper or range)** | **Cost-consequence analysis:**  **Cost‑neutral value** | **Cost-effectiveness analysis:**  **Value to achieve ICER of £20,000/QALY** |
| --- | --- | --- | --- |
| Cost - Drug pain therapy with SCS  (6 months) | £2,012  (£0 to £8,412) | £3,919 | £11,183 |
| Cost - Drug pain therapy CMM alone (6 months) | £3,167  (£0 to £8,412) | £1,239 | -£6,102 |
| Year 3+ explant rate: 10kHz‑SCS | 3.2%  (0.0% to 15.8%) | 8.8% | 15.5% |
| Cost - IPG system: 10kHz‑SCS | £16,648  (£13,116 to £21,421) | £20,185 | £33,655 |
| Cost - IPG system: RLF-SCS | £17,422  (£13,726 to £22,418) | £13,547 | –£1,211 |
| Year 3+ explant rate: RLF-SCS | 3.2%  (0.0% to 15.8%) | -1.6%^†^ | -6.0% |
| Device longevity: 10kHz-SCS | 10 (8 to 25) | 6.75^‡^ | NA |
| Device longevity: RLF-SCS | 10 (8 to 25) | 15.25^‡^ | NA |
| Year 1 explant rate: RLF-SCS | 11.1%  (4.3% to 18.0%) | -8.8%^†^ | Not a top 10 driver |
| Year 2 explant rate: RLF-SCS | 9.7%  (2.9% to 16.6%) | -13.1%^†^ | Not a top 10 driver |
| Cost - Non-drug pain therapy CMM alone (6 months) | £956  (£0 to £1,157) | Not a top 10 driver | –£8,314 |
| Optimal pain relief: RLF-SCS | 54.4%  (43.5% to 65.2%) | Not a top 10 driver | NA |

Abbreviations: 10kHz‑SCS, 10kHz high-frequency spinal cord stimulation; CI, confidence interval; CMM, conventional medical management; ICER, incremental cost‑effectiveness ratio; IPG, interventional procedure guidance; RLF-SCS, rechargeable low-frequency spinal cord stimulation; QALY, quality adjusted life year; SCS, spinal cord stimulation.

† These values are not the values for cost-neutrality. Due to the quarterly cycle length it is not possible to have a zero‑cost difference. The figures reported indicate the point at which 10kHz‑SCS is more costly.

‡ These values are negative and would not occur in reality, i.e. they are outside a plausible range.
