# Supplementary material for: High-frequency 10 kHz Spinal Cord Stimulation for Chronic Back and Leg Pain: Cost-consequence and Cost-effectiveness Analyses
Source: Clin J Pain. 2020 Aug 4;36(11):852–61. doi: 10.1097/AJP.0000000000000866 (PMC7671822; doi:10.1097/AJP.0000000000000866)
Supplement: SUPPLEMENTARY MATERIAL [file ajp-36-852-s007.docx]

## Multi‑way sensitivity analyses

### **Back pain response**

To be consistent with the model submitted to the National Institute for Health and Care Excellence (NICE) for Technology Appraisal 159 in 2008 (TAG159) [[1](#_ENREF_1)], in the base‑case analysis pain relief is assessed using leg pain response, from the SENZA-RCT [[2](#_ENREF_2)]. The SENZA-RCT also reports pain relief assessed using back pain response (primary endpoint). The values for efficacy that populate the decision tree element of the model when utilising pain relief assessed as back pain response rather than leg pain response are outlined in **e-Table 7**.

**e-Table 7 - Alternative variables for the decision tree: Pain relief assessed as back pain response**

| **Model parameter** | **Base‑case value** | **Source** |
| --- | --- | --- |
| *Optimal pain relief (back pain, 6 months)* | | |
| 10kHz‑SCS | 76.4% | Kapural et al. (2015) [[2](#_ENREF_2)] |
| NRLF-SCS/RLF-SCS | 51.9% | Kapural et al. (2015) [[2](#_ENREF_2)] |
| **Calculated values from the SENZA-RCT** | | |
| *Optimal pain relief without complications* | | |
| 10kHz‑SCS | 50.6% | Calculated from SENZA-RCT |
| NRLF-SCS/RLF-SCS | 33.3% | Calculated from SENZA-RCT |
| *Optimal pain relief with complications* | | |
| 10kHz‑SCS | 25.8% | Calculated from SENZA-RCT |
| NRLF-SCS/RLF-SCS | 18.6% | Calculated from SENZA-RCT |
| *Sub-optimal pain relief without complications* | | |
| 10kHz‑SCS | 15.6% | Calculated from SENZA-RCT |
| NRLF-SCS/RLF-SCS | 30.9% | Calculated from SENZA-RCT |
| *Sub-optimal pain relief with complications* | | |
| 10kHz‑SCS | 8.0% | Calculated from SENZA-RCT |
| NRLF-SCS/RLF-SCS | 17.2% | Calculated from SENZA-RCT |

Abbreviations: 10kHz‑SCS, 10kHz high frequency spinal cord stimulation; NRLF-SCS, non‑rechargeable low‑frequency spinal cord stimulation; RCT, randomised controlled trial; RLF‑SCS, rechargeable low‑frequency spinal cord stimulation.
