# Supplementary material for: High-frequency 10 kHz Spinal Cord Stimulation for Chronic Back and Leg Pain: Cost-consequence and Cost-effectiveness Analyses
Source: Clin J Pain. 2020 Aug 4;36(11):852–61. doi: 10.1097/AJP.0000000000000866 (PMC7671822; doi:10.1097/AJP.0000000000000866)
Supplement: SUPPLEMENTARY MATERIAL [file ajp-36-852-s008.docx]

**e-Table 8 - Results of scenario using back pain response as alternative to leg pain response from SENZA-RCT**

|  | **Total cost per patient** | **Cost saving with 10kHz‑SCS therapy** |
| --- | --- | --- |
| 10kHz-SCS | £87,400 | - |
| NRLF-SCS | £95,156 | £7,755 |
| RLF-SCS | £92,196 | £4,795 |

Abbreviations: 10kHz‑SCS, 10kHz high frequency spinal cord stimulation; NRLF-SCS, non‑rechargeable low‑frequency spinal cord stimulation; RCT, randomised controlled trial; RLF‑SCS, rechargeable low‑frequency spinal cord stimulation.

As it is assumed there is no differential between 10kHz‑SCS therapy and NRLF-SCS/RLF-SCS for the costs of optimal and sub-optimal pain relief, the use of an alternative measure of efficacy has no impact on the cost saving. Fewer patients will reach optimal pain relief when assessed via back pain response; however, 10kHz‑SCS therapy still results in cost savings and more patients have optimal pain relief compared to NRLF-SCS/RLF-SCS.
