# Supplementary material for: High-frequency 10 kHz Spinal Cord Stimulation for Chronic Back and Leg Pain: Cost-consequence and Cost-effectiveness Analyses
Source: Clin J Pain. 2020 Aug 4;36(11):852–61. doi: 10.1097/AJP.0000000000000866 (PMC7671822; doi:10.1097/AJP.0000000000000866)
Supplement: SUPPLEMENTARY MATERIAL [file ajp-36-852-s009.docx]

### **Pain relief assessed at 3, 12 and 24 months**

Again, for consistency with the NICE TAG159 model [[1](#_ENREF_1)], in the base‑case analysis pain relief was assessed at 6-months using data from the SENZA-RCT [[2](#_ENREF_2)]. However, pain relief assessment was also repeated at 3 and 12 months in the original NICE TAG159 model [[1](#_ENREF_1)], and at 24 months in the SENZA-RCT [[3](#_ENREF_3)]. A scenario analysis was conducted to consider the impact of assessing pain relief at 3, 12 and 24 months (**e-Table 9**). Minor modifications were made to the current model; amending the decision tree to reflect either 3, 12 or 24 months and modifying the Markov section to allow entry at the appropriate associated time points.

**e-Table 9 - Alternative variables for the decision tree: Pain relief assessed at 3, 12 and 24 months**

| **Model parameter** | **Base‑case value** | **Source** |
| --- | --- | --- |
| *Optimal pain relief (leg pain, 3 months)* | | |
| 10kHz‑SCS | 83.1% | Kapural et al. (2015) [[2](#_ENREF_2)] |
| NRLF-SCS/RLF-SCS | 55.0% | Kapural et al. (2015) [[2](#_ENREF_2)] |
| *Optimal pain relief (leg pain, 12 months)* | | |
| 10kHz‑SCS | 78.7% | Kapural et al. (2015) [[2](#_ENREF_2)] |
| NRLF-SCS/RLF-SCS | 51.3% | Kapural et al. (2015) [[2](#_ENREF_2)] |
| *Optimal pain relief (leg pain, 24 months)* | | |
| 10kHz‑SCS | 72.9% | Kapural et al. (2016)[[3](#_ENREF_3)] |
| NRLF-SCS/RLF-SCS | 49.3% | Kapural et al. (2016) [[3](#_ENREF_3)] |

Abbreviations: 10kHz‑SCS, 10kHz high frequency spinal cord stimulation; NRLF-SCS, non‑rechargeable low‑frequency spinal cord stimulation; RLF‑SCS, rechargeable low‑frequency spinal cord stimulation.
