# Supplementary material for: High-frequency 10 kHz Spinal Cord Stimulation for Chronic Back and Leg Pain: Cost-consequence and Cost-effectiveness Analyses
Source: Clin J Pain. 2020 Aug 4;36(11):852–61. doi: 10.1097/AJP.0000000000000866 (PMC7671822; doi:10.1097/AJP.0000000000000866)
Supplement: SUPPLEMENTARY MATERIAL [file ajp-36-852-s010.docx]

The relatively small changes in efficacy over time result in only small changes in the cost differentials (**e-Table 10**).

**e-Table 10 - Results of scenario using pain relief assessment at 3, 12 and 24 months**

|  | **Total cost per patient** | **Cost saving with 10kHz‑SCS therapy** |
| --- | --- | --- |
| *3 months* | | |
| 10kHz‑SCS | £87,426 | - |
| NRLF-SCS | £95,158 | £7,732 |
| RLF-SCS | £92,282 | £4,856 |
| *12 months* | | |
| 10kHz‑SCS | £87,390 | - |
| NRLF-SCS | £95,182 | £7,793 |
| RLF-SCS | £92,050 | £4,661 |
| *24 months* | | |
| 10kHz‑SCS | £87,544 | - |
| NRLF-SCS | £95,448 | £7,904 |
| RLF-SCS | £91,961 | £4,418 |

Abbreviations: 10kHz‑SCS, 10kHz high frequency spinal cord stimulation; NRLF-SCS, non‑rechargeable low‑frequency spinal cord stimulation; RLF‑SCS, rechargeable low‑frequency spinal cord stimulation.
