# Supplementary material for: High-frequency 10 kHz Spinal Cord Stimulation for Chronic Back and Leg Pain: Cost-consequence and Cost-effectiveness Analyses
Source: Clin J Pain. 2020 Aug 4;36(11):852–61. doi: 10.1097/AJP.0000000000000866 (PMC7671822; doi:10.1097/AJP.0000000000000866)
Supplement: SUPPLEMENTARY MATERIAL [file ajp-36-852-s011.docx]

### **Alternative system costs**

The cost of reimplantation for NRLF-SCS and RLF-SCS is assumed to be lower than the cost of permanent implantation in the Taylor et al. (2010) analysis [[4](#_ENREF_4)]. **e-Table 11** presents the results utilising the alternative costs for reimplantation. In this analysis, the cost of reimplantation is assumed to be marginally cheaper than the cost of permanent implantation and the permanent implantation cost of RLF-SCS is conservatively assumed to be equal to 10kHz‑SCS therapy.

**e-Table 11 - Alternative system cost results**

|  | Total cost per patient | Cost saving with 10kHz‑SCS therapy |
| --- | --- | --- |
| 10kHz‑SCS | £86,354 | - |
| NRLF-SCS | £94,152 | £7,798 |
| RLF-SCS | £90,363 | £4,009 |

Abbreviations: 10kHz-SCS, 10kHz high frequency spinal cord stimulation; NRLF-SCS, non‑rechargeable low‑frequency spinal cord stimulation; RLF SCS, rechargeable low‑frequency spinal cord stimulation.

This analysis results in slightly lower absolute costs due to the reduction in reimplantation costs; 10kHz‑SCS therapy still remains cost‑saving, even with the conservative assumption of the permanent implantation cost of RLF-SCS being equal to 10kHz‑SCS therapy.
