# Supplementary material for: High-frequency 10 kHz Spinal Cord Stimulation for Chronic Back and Leg Pain: Cost-consequence and Cost-effectiveness Analyses
Source: Clin J Pain. 2020 Aug 4;36(11):852–61. doi: 10.1097/AJP.0000000000000866 (PMC7671822; doi:10.1097/AJP.0000000000000866)
Supplement: SUPPLEMENTARY MATERIAL [file ajp-36-852-s012.docx]

### **Time horizon**

For consistency with the model and assumptions accepted by NICE for TAG159 [[1](#_ENREF_1)], the base‑case analysis utilises a 15-year time horizon. It is felt this is appropriate in the base‑case given the chronic nature of the condition and the longevity of the devices, particularly those utilising a rechargeable battery. The Markov model structure allows us to present the incremental cost at various time-points as shown in **e-Figure 1**. In the base‑case analysis, the 10kHz‑SCS therapy implantation cost is marginally less than RLF-SCS and therefore 10kHz-SCS therapy is cost‑saving from the start of the analysis, additional cost-savings are realised over time due to the superior explant rates associated with 10kHz‑SCS therapy.

The NRLF-SCS implantation cost is less than 10kHz‑SCS therapy; therefore NRLF‑SCS remains less costly until the first NRLF‑SCS device replacement. However, after this point 10kHz‑SCS therapy is cost‑saving.

**e-Figure 1 - Incremental cost difference over time**


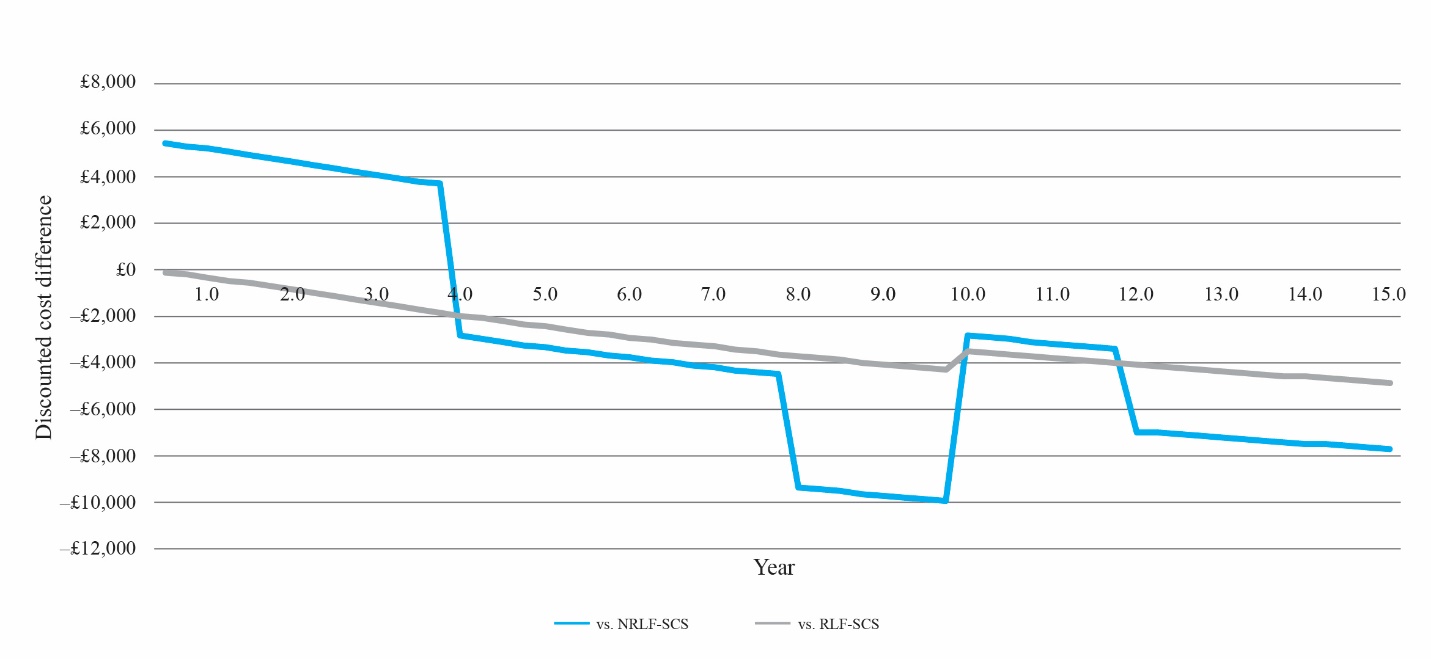


Abbreviations: NRLF-SCS, non‑rechargeable low‑frequency spinal cord stimulation; RLF SCS, rechargeable low‑frequency spinal cord stimulation.
